# Supplementary material for: MetaRibo-Seq measures translation in microbiomes
Source: Nat Commun. 2020 Jun 29;11:3268. doi: 10.1038/s41467-020-17081-z (PMC7324362; doi:10.1038/s41467-020-17081-z)
Supplement: Supplementary file 10 — Supplementary Data 7 [file 41467_2020_17081_MOESM10_ESM.zip › File2/Confidence_High_Taxonomy/30279_out.krona.html]

Javascript must be enabled to view this page.

members
magnitude
magnitudeUnassigned
count
unassigned
taxon
rank

30279\_out

82

2
superkingdom
82

1239
82
phylum

82
class
186801

186802
order
4
82

SRS018427\_contig\_number\_contig-100\_7146.96969SRS047014\_contig\_number\_contig-100\_704.169130SRS1041033\_contig\_number\_contig-100\_34610.68205SRS149784\_contig\_number\_contig-100\_9226.50801

1
family
186806

1
genus
1730


SRS143342\_contig\_number\_13604
1
species
1262889

541000
family
2

216851
genus
1

853
1
species

SRS019068\_contig\_number\_16436

1
genus
1263

165186
1
species

SRS142503\_contig\_number\_50103

family
18
186803


SRS144714\_contig\_number\_4774
1
species
39491

1898203
species
16

SRS014855\_contig\_number\_15173SRS015065\_contig\_number\_2314SRS015431\_contig\_number\_10104SRS015579\_contig\_number\_contig-100\_5207.111143SRS016095\_contig\_number\_contig-100\_254.121508SRS016989\_contig\_number\_contig-100\_17152.53674SRS019496\_contig\_number\_contig-100\_27063.27064SRS045004\_contig\_number\_13002SRS053335\_contig\_number\_18577SRS063040\_contig\_number\_contig-100\_18756.151540SRS065504\_contig\_number\_22824SRS075984\_contig\_number\_19843SRS078419\_contig\_number\_contig-100\_965.82296SRS144135\_contig\_number\_34081SRS148319\_contig\_number\_6780SRS148424\_contig\_number\_30527

1
genus
830

45851
1
species

SRS098644\_contig\_number\_4488

31979
family
57

1485
57
genus


SRS050941\_contig\_number\_15302
1
species
2293035

2293015

SRS011134\_contig\_number\_46924SRS011302\_contig\_number\_9308SRS015663\_contig\_number\_32140SRS019267\_contig\_number\_contig-100\_10525.41568SRS019601\_contig\_number\_contig-100\_12718.92480SRS020869\_contig\_number\_contig-100\_31810.116530SRS021948\_contig\_number\_22518SRS022071\_contig\_number\_28918SRS022609\_contig\_number\_29558SRS049900\_contig\_number\_contig-100\_8787.66485SRS051031\_contig\_number\_24863SRS075821\_contig\_number\_20580SRS077502\_contig\_number\_16483SRS077552\_contig\_number\_27981SRS1041038\_contig\_number\_14882SRS1055067\_contig\_number\_14359SRS143466\_contig\_number\_contig-100\_42.142635SRS144362\_contig\_number\_30948
species
18


SRS893270\_contig\_number\_12342
species
1
411489

1896990
species
12

SRS014313\_contig\_number\_5104SRS014613\_contig\_number\_14156SRS014979\_contig\_number\_34153SRS024132\_contig\_number\_20685SRS049773\_contig\_number\_32580SRS076756\_contig\_number\_21740SRS077231\_contig\_number\_13044SRS097889\_contig\_number\_3371SRS104485\_contig\_number\_4260SRS105153\_contig\_number\_37724SRS1055022\_contig\_number\_24486SRS142923\_contig\_number\_11194

1262828
20
species

SRS013098\_contig\_number\_13264SRS013687\_contig\_number\_23088SRS015854\_contig\_number\_135SRS016954\_contig\_number\_contig-100\_11446.40584SRS017821\_contig\_number\_24240SRS018623\_contig\_number\_5288SRS019161\_contig\_number\_36473SRS020328\_contig\_number\_contig-100\_6434.78354SRS042966\_contig\_number\_contig-100\_3759.104895SRS052697\_contig\_number\_25921SRS063985\_contig\_number\_30546SRS064276\_contig\_number\_39309SRS104197\_contig\_number\_contig-100\_194.6353SRS142542\_contig\_number\_20015SRS143523\_contig\_number\_9704SRS143895\_contig\_number\_34824SRS147766\_contig\_number\_13069SRS147977\_contig\_number\_6873SRS148159\_contig\_number\_36096SRS971275\_contig\_number\_8660

5
species

SRS013965\_contig\_number\_contig-100\_1631.196905SRS015694\_contig\_number\_contig-100\_77.137990SRS077194\_contig\_number\_28784SRS143780\_contig\_number\_14961SRS893187\_contig\_number\_6108
2293016
